# Increased prevalence of kidney cysts in individuals carrying heterozygous *COL4A3* or *COL4A4* pathogenic variants

**DOI:** 10.1093/ndt/gfae031

**Published:** 2024-02-05

**Authors:** Mónica Furlano, Melissa Pilco-Teran, Marc Pybus, Víctor Martínez, Miriam Aza-Carmona, Asunción Rius Peris, Vanessa Pérez-Gomez, Gerson Berná, Jaime Mazon, Jonathan Hernández, Leonor Fayos de Arizón, Elizabet Viera, Ignasi Gich, Hugo Vergara Pérez, Elena Gomá-Garcés, José Luis Albero Dolon, Elisabet Ars, Roser Torra

**Affiliations:** Inherited Kidney Diseases, Nephrology Department, Fundació Puigvert, Institut de Recerca Sant Pau, Department of Medicine, Universitat Autonoma de Barcelona (UAB), Barcelona, Spain; Inherited Kidney Diseases, Nephrology Department, Fundació Puigvert, Institut de Recerca Sant Pau, Department of Medicine, Universitat Autonoma de Barcelona (UAB), Barcelona, Spain; Molecular Biology Laboratory, Fundació Puigvert, Institut de Recerca Sant Pau, Barcelona, Spain; Nephrology Department, Hospital Universitario Virgen de la Arrixaca, Arrixaca, Spain; Molecular Biology Laboratory, Fundació Puigvert, Institut de Recerca Sant Pau, Barcelona, Spain; Nephrology Department, Hospital General Universitario de Castellón, Castellón, Spain; Nephrology Department, Hospital Universitario Fundación Jiménez Díaz, Madrid, Spain; Nephrology Department, Fundació Puigvert, Barcelona, Spain; Nephrology Department, Hospital de Valdecilla, Santander, Spain; Radiology Department, Fundació Puigvert, Barcelona, Spain; Nephrology Department, Fundació Puigvert, Barcelona, Spain; Nephrology Department, Fundació Puigvert, Barcelona, Spain; Consorcio de Investigación Biomédica en Red de Epidemiología y Salud Pública (CIBERESP), Barcelona, Spain; Nephrology Department, Hospital General Universitario de Castellón, Castellón, Spain; Nephrology Department, Hospital Universitario Fundación Jiménez Díaz, Madrid, Spain; Nephrology Department, Hospital Universitario Virgen de la Arrixaca, Arrixaca, Spain; Molecular Biology Laboratory, Fundació Puigvert, Institut de Recerca Sant Pau, Barcelona, Spain; Inherited Kidney Diseases, Nephrology Department, Fundació Puigvert, Institut de Recerca Sant Pau, Department of Medicine, Universitat Autonoma de Barcelona (UAB), Barcelona, Spain

**Keywords:** Alport syndrome, *COL4A3*, *COL4A4*, kidney cysts, type IV collagen nephropathy

## Abstract

**Background:**

Clinical variability among individuals with heterozygous pathogenic/likely pathogenic (P/LP) variants in the *COL4A3*/*COL4A4* genes (also called autosomal dominant Alport syndrome or *COL4A3/COL4A4*-related disorder) is huge; many individuals are asymptomatic or show microhematuria, while others may develop proteinuria and chronic kidney disease (CKD). The prevalence of simple kidney cysts (KC) in the general population varies according to age, and patients with advanced CKD are prone to have them. A possible association between heterozygous *COL4A3, COL4A4* and *COL4A5* P/LP variants and KC has been described in small cohorts. The presence of KC in a multicenter cohort of individuals with heterozygous P/LP variants in the *COL4A3*/*COL4A4* genes is assessed in this study.

**Methods:**

We evaluated the presence of KC by ultrasound in 157 individuals with P/LP variants in *COL4A3* (40.7%) or *COL4A4* (53.5%) without kidney replacement therapy. The association between presence of KC and age, proteinuria, estimated glomerular filtration rate (eGFR) and causative gene was analyzed. Prevalence of KC was compared with historical case series in the general population.

**Results:**

Half of the individuals with P/LP variants in *COL4A3*/*COL4A4* showed KC, which is a significantly higher percentage than in the general population. Only 3.8% (6/157) had cystic nephromegaly. Age and eGFR showed an association with the presence of KC (*P* < .001). No association was found between KC and proteinuria, sex or causative gene.

**Conclusions:**

Individuals with *COL4A3*/*COL4A4* P/LP variants are prone to develop KC more frequently than the general population, and their presence is related to age and to eGFR. Neither proteinuria, sex nor the causative gene influences the presence of KC in these individuals.

## Graphical Abstract

**Graphical Abstract:**
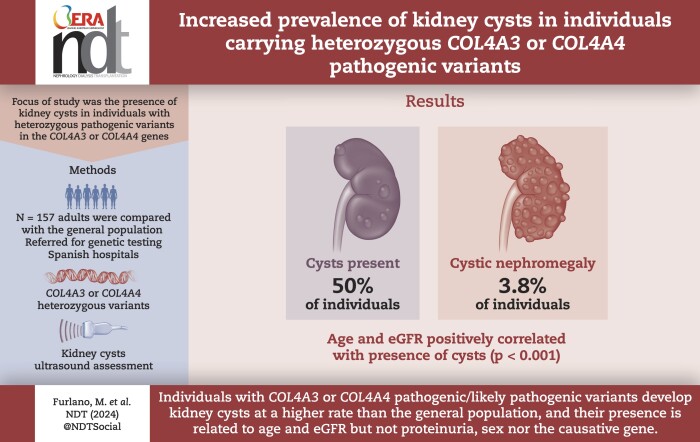

KEY LEARNING POINTS **What was known:**

- Insufficient information is available in the literature on the prevalence of kidney cysts (KC) in individuals with pathogenic/likely pathogenic (P/LP) variants in the *COL4A3/COL4A4* genes.

**This study adds:**

- This is the largest cohort published to date of individuals with P/LP variants in the *COL4A3/COL4A4* genes and ultrasound assessment to evaluate the presence of KC.
- It demonstrates that the presence of KC in these individuals is higher than in age-matched general population.
- The prevalence of KC is associated with age and estimated glomerular filtration rate.

**Potential impact:**

- This study demonstrates that there is an association between P/LP variants in the *COL4A3/COL4A4* genes and presence of KC.
- There is currently insufficient evidence to recommend testing for *COL4A3*–*COL4A5* variants in cystic kidney panels but it may be considered. The clinical impact of KC in these individuals seems to be negligible.

## INTRODUCTION

Kidney cysts (KC) result from genetic and nongenetic processes and occur in multiple diseases as well as in healthy individuals. The most common type of radiologically evident KC in adults are simple cysts [1]. Simple KC are commonly observed in normal-sized kidneys, with an increasing incidence with age [2, 3]. Although the volume of the kidney is usually normal, these cysts can exceptionally increase the total kidney volume.

KC are benign, asymptomatic lesions that rarely require treatment [4–6]. The clinical concerns that usually arise regarding KC are (i) whether malignancy can be ruled out and (ii) whether the KC can be considered simple cysts or part of a disease [7].

The prevalence of simple KC varies according to the studied series, for example from 0% to 10% in individuals younger than 20 years [8], from 5% to 20% in those aged between 20 and 50 years, and from 30% to 50% in those older than 60 years [1, 2, 9]. Bilateral KC are much less common and seem to be rare in subjects <50 years of age [1, 9–11]. It has also been reported that KC are more common in men than in women [1].

The most frequent inherited cystic kidney disease is autosomal dominant polycystic kidney disease (ADPKD) [12, 13]. It is characterized by multiple bilateral fluid-filled cysts, progressively increased kidney volume and irreversible renal insufficiency [13, 14]. However, KC also occur in other inherited kidney diseases, such as autosomal recessive polycystic kidney disease, autosomal dominant tubulointerstitial kidney disease, nephronophthisis, tuberous sclerosis complex, von Hippel–Lindau disease, hereditary angiopathy with nephropathy, aneurysms and muscle cramps (HANAC) syndrome, autosomal dominant liver disease, *HNF1B* nephropathy and oral–facial–digital syndrome [15].

Acquired cystic kidney disease (ACKD) is defined as the presence of three or more cysts in each kidney in patients with advanced cystic kidney disease (CKD) or on kidney replacement therapy (KRT), and small or normal-sized kidneys. It seems to be caused by prolonged uremia and may represent aberrant compensatory tubular growth as its prevalence increases as kidney function declines [16–19]. The reported prevalence of ACKD is as low as 7% in predialysis patients and as high as 80% in patients who have been on KRT for 10 years [17].

Pathogenic/likely pathogenic (P/LP) sequence variants in the *COL4A3/COL4A4* genes cause a condition that is under-recognized (ranging from completely normal urine sediment to microhematuria, proteinuria and renal insufficiency), poorly understood and not yet well classified [20–22]. Recently, the prevalence of heterozygous P/LP variants in *COL4A3/COL4A4* has been reported to be as high as 1 in 106 of the population [23]. Although we have previously published a cohort of patients with this condition and referred to them as having autosomal dominant Alport syndrome [24], this name is probably no longer appropriate, given the high prevalence in the population and the lack of extrarenal or even renal manifestations in most individuals. Also, from a medical-ethical point of view, it seems inappropriate to consider individuals without any clinical features as “patients.” Further insight into this entity is needed before an appropriate name can be given [25–28].

Small cohorts of individuals with collagen type IV nephropathy and KC have been reported [29, 30], which raises the possibility of a link between P/LP variants in these genes and KC.

Here we present the largest series published to date, which demonstrates an increased prevalence of KC in individuals with heterozygous P/LP variants in the *COL4A3/COL4A4* genes.

## MATERIALS AND METHODS

A multicenter cohort, based in part on a previously reported cohort of patients with heterozygous P/LP variants in the *COL4A3/COL4A4* genes [24] and kidney ultrasound, was retrospectively studied (Supplementary data, Table S1). Individuals were referred for genetic testing in the context of families with autosomal dominantly inherited hematuria ± proteinuria and/or CKD, or index patients with an abnormal glomerular basement membrane. It consisted of a total of 157 adults (>18 years old) without KRT, belonging to 104 families from different Spanish hospitals [24].

The following clinical data were recorded at the time of kidney ultrasound: age, sex, serum creatinine, estimated glomerular filtration rate (eGFR; measured by CKD Epidemiology Collaboration), presence of hematuria, presence of lithiasis, and proteinuria measured by urinary protein/creatinine ratio and divided into four categories: no proteinuria, albuminuria (30–300 mg/gCr), non-nephrotic proteinuria (300–3500 mg/gCr) and nephrotic-range proteinuria (>3500 mg/gCr). Kidney length and number of KC in each kidney were recorded. Nephromegaly was considered when the kidney size was >13 cm by ultrasound [31–33].

One plus (1+) KC were defined as the presence of at least one cyst in one kidney. Three plus (3+) KC were considered present when three or more KC were observed, either unilaterally or bilaterally.

Data from a published general population cohort that assessed the prevalence and size of KC using magnetic resonance imaging (MRI) was used to compare the presence of KC [3]. This cohort comprised 2063 participants in the Pomeranian Health Study conducted in a general German population sample between 2008 and 2012 [3].

This study was approved by the Fundació Puigvert institutional review board and the different healthcare provider institutional review boards. All participants signed their informed consent to participation in the study.

### Genetic testing

Genetic testing had been performed by next generation sequencing of a kidney-disease gene panel containing *COL4A3, COL4A4* and *COL4A5* genes, along with other genes associated to inherited kidney diseases [24, 34, 35] (Supplementary data, Table S2) in 106 individuals. In all of them, 85 genes related to KC were thoroughly analyzed to discard any other genetic cause of KC. In the remaining 51 individuals, targeted familial variant analysis was performed by Sanger or multiplex ligation-dependent probe amplification analysis (MLPA). Variants were classified using American College of Medical Genetics and Genomics/Association for Molecular Pathology guidelines [36], and P or LP variants were considered causative. Segregation analysis was performed, in patients with available family members, by Sanger sequencing or MLPA. A digenic/complex inheritance pattern was considered present when two P/LP variants were identified in both *COL4A3*/*COL4A4* genes.

### Statistical analysis

Categorical variables are expressed as percentages and frequencies and were compared using Pearson's chi-squared test. Quantitative variables with normal distribution are expressed by measures of central tendency, including mean and standard deviation, and were compared using Student's *t*-test. The logistic regression analysis was modelled with age and eGFR as dependent variables. The general population comparison analysis was performed using the two-proportions z-test. A *P*-value <.05 was considered statistically significant.

All statistical analyses were conducted in R (R version 4.1.2).

## RESULTS

### Baseline characteristics of the study cohort

The cohort included a total of 157 individuals from 104 families (56.6% women 89/157) with P/LP variants in the *COL4A3* (40.1%; 64/157) or *COL4A4* (54.1%; 86/157) gene (Table 1). Nine individuals showed a digenic inheritance (5.7%; 9/157). Hematuria was present in 92.3% (145/157) and albuminuria or proteinuria were present in 63.7% (100/157) of the patients. Only 12.1% (19/157) of the patients exhibited a severe eGFR category (G4 and G5). The mean age at the time of kidney ultrasound was 47.8 ± 13.6 years old and the mean eGFR was estimated at 73.0 ± 32.5 mL/min/1.73 m^2^. Average right and left kidney lengths fell within the expected normal distribution (104.2 ± 13.1 and 105.3 ± 12.1 mm, respectively). Nephromegaly was observed in six patients, and three of them showed bilateral nephromegaly with 3+ KC (Table 2). The mean kidney lengths of the right and left kidneys of these patients with nephromegaly were 143.83 ± 16.31 and 134.17 ± 11.99 mm respectively. Only three patients showed lithiasis at ultrasound.

**Table 1:** Baseline characteristics of the cohort.

Cohort study	*N* = 157 [*n*/*N* (%)]
Sex (female)	92/157 (58.6)
Hematuria	145/156 (92.9)
Causative gene	
*COL4A3*	64/157 (40.7)
*COL4A4*	84/157 (53.5)
Digenic (*COL4A3* and *COL4A4*)	9/157 (5.7)
eGFR category	
G1	58/157 (36.9)
G2	38/157 (24.2)
G3a	19/157 (12.1)
G3b	23/157 (14.6)
G4	14/157 (8.9)
G5	5/157 (3.2)
Proteinuria/creatinine ratio	
No albuminuria (<30 mg/gCr)	57/157 (36.3)
Albuminuria (30–300 mg/gCr)	52/157 (33.12)
Non-nephrotic (300–3500 mg/gCr)	43/157 (27.3)
Nephrotic (>3500 mg/gCr)	5/157 (3.2)
Lithiasis	3/142 (2.1)
Presence of 1+ KC	
Either kidney	84/157 (53.5)
Bilateral	53/157 (33.7)
Presence of 3+ KC	
Either kidney	44/157 (28.0)
Bilateral	41/157 (26.1)
**Cohort study**	***N* = 157 (mean ± SD)**
Kidney length	
Right kidney (mm)	104.2 ± 13.1
Left kidney (mm)	105.3 ± 12.1
Age (years)	47.8 ± 13.6
eGFR (mL/min/1.73 m^2^)	73.0 ± 32.5

**Table 2:** Characteristics of patients with nephromegaly (kidney length >130 mm) and KC.

Patient	Sex	Age (years)	eGFR (mL/min/1.73 m^2^)	CKD category	PCR (mg/gCr)	RKS (mm)	LKS (mm)	Number RKC	Number LKC
07–0440	M	37	101	1	207	132	132	0	0
12–508	M	51	38	3b	761	170	150	3+	3+
20–0003	F	38	56	3a	284	139	145	3+	3+
20–0517	M	46	105	1	15	133	125	1	1
21–0463	M	22	125	1	40	131	118	0	1
22–0640	M	65	54	3a	65	158	135	3+	3+

F: female; LKC: left kidney cysts; LKS: left kidney size; M: male; PCR: protein/creatinine ratio; RKC: right kidney cysts; RKS: right kidney size.

### Association of KC and clinical variables

KC were present in 53.5% of the patients (84/157) and bilateral KC in 63% (53/84) of those. Besides, 28.0% (44/157) of the patients showed 3 or more KC in either kidney. The percentage of individuals by age and eGFR strata and the number of KC are shown in Supplementary data, Table S3. In a univariate association analysis, only older age and lower eGFR were found to be statistically associated with the presence of 1+ KC (*P* < .001) (Table 3), as well as the presence of 3+ KC (*P* < .001) (Table 4). Both variables were then included in a multivariate logistic regression analysis that showed a statistically significant association of age with the presence of 1+ KC (*P* = .003), and of age and eGFR with the presence of 3+ KC (*P* = .008 and *P* = .001, respectively) (Table 5).

**Table 3:** Association analysis for presenting 1+ KC in either kidney.

	1+ KC	No KC	*P*-value
Sex (female)	55.9 (47/84)	61.6 (45/73)	.57
Hematuria	91.7 (77/84)	93.1 (67/72)	.98
Causative gene (*COL4A3*)	43.2 (35/81)	43.3 (29/67)	1
Lithiasis	2.8 (2/72)	1.4 (1/70)	1
Age (years)	53.3 ± 12.9	41.5 ± 11.6	<.001
eGFR (mL/min/1.73 m^2^)	62.3 ± 31.2	85.3 ± 29.7	<.001
PCR (mg/gCr)	488.5 ± 946.6	509.0 ± 1069.4	.89

Data are presented as % (*n*/*N*) or mean ± SD.

PCR: protein/creatinine ratio.

**Table 4:** Association analysis for presenting 3+ KC in either kidney.

	3+ KC	<3 KC	*P*-value
Sex (female)	52.3 (23/44)	61.1 (69/113)	.41
Hematuria	93.2 (41/44)	91.9 (103/112)	1
Causative gene (*COL4A3*)	38.1 (16/42)	45.3 (48/106)	.54
Lithiasis	0.0 (0/37)	2.8 (3/105)	.71
Age (years)	56.3 ± 11.2	44.4 ± 13.1	<.001
eGFR (mL/min/1.73 m^2^)	51.1 ± 29.2	81.5 ± 29.7	<.001
PCR (mg/gCr)	664.2 ± 1184.1	433.3 ± 919.8	.24

Data are presented as % (*n*/*N*) or mean ± SD.

PCR: protein/creatinine ratio.

**Table 5:** Logistic regression analysis for presenting 1+ KC or 3+ KC in either kidney.

	1+ KC (OR, *P*-value)	3+ KC (OR, *P*-value)
Age (years)	1.06, *P* < .003	1.05, *P* < .008
eGFR (mL/min/1.73 m^2^)	0.99, *P* = .09	0.97, *P* < .001

OR: odds ratio.

### Causative gene and presence of KC

Among patients with P/LP variants in *COL4A3*, 54.7% (35/64) presented with KC while among patients with P/LP variants in *COL4A4*, 54.8% (46/84) presented with KC; there was no statistically significant association between gene and the presence of KC.

### Comparison with a cohort from the general population

Data from Mensel *et al*. [3] were used to perform a sex- and age-matched general population comparison analysis of 1+ KC prevalence estimates found in this study cohort (Table 6). Males with *COL4A3* or *COL4A4* P/LP variants showed a prevalence of 58.1% (36/62) compared with 33.7% (341/1011) in the general population (*P* < .0001). Females showed a prevalence of 52.1% (48/92) compared with 21.2% (223/1052) in the general population (*P* < .0001). Additionally, an age-matched analysis showed higher prevalence across all age groups within each sex, but only reached a statistically significant association in the group age of 50- to 59-year-old males, and in the group age of 30- to 69-year-old females.

**Table 6:** Comparison of prevalence in presenting 1+ KC in either kidney by sex and age in this cohort study and a cohort from the general population.

Age (years)	20–29	30–39	40–49	50–59	60–69	>70	Total
Males							
This cohort study	1/4 (25)	4/10 (40)	8/17 (47)	11/17 (64.7)	8/10 (80)	4/4 (100)	36/62 (58.1)
General population^a^	13/92 (14.1)	29/146 (19.8)	62/239 (25.9)	84/236 (35.5)	86/177 (48.6)	67/121 (55.3)	341/1011 (33.7)
*P*-value	1	.267	.108	.030	.107	.277	<.001
Females							
This cohort study	2/7 (28.6)	7/23 (30.4)	9/20 (45)	15/25 (60)	11/13 (84.6)	4/4 (100)	48/92 (52.1)
General population^a^	5/70 (7.1)	17/151 (11.2)	40/263 (15.2)	50/267 (18.7)	71/209 (33.9)	40/92 (43.4)	223/1052 (21.2)
*P*-value	.233	.031	.002	.0001	.0007	.087	<.001

Data are presented as *n*/*N* (%).

^a^From Mensel *et al*. [3] Three patients under 20 years were not included in this analysis (all without kidney cysts).

## DISCUSSION

Some small cohort studies have reported the presence of KC in individuals with *COL4A3*/*COL4A4* [30]. It has also been shown that KC coexist with focal segmental glomerulosclerosis, suggesting that KC can be caused by a severe defect in the glomerular basement membrane [37, 38]. In addition, KC have been described in a small cohort of 32 patients with kidney biopsy-proven thin basement membrane disease (TBMD) without genetic testing, showing a positive correlation with proteinuria; in contrast, no KC were detected in patients with TBMD with normal kidney function and without proteinuria [29]. It has been hypothesized that KC may result from distension of basement membranes weakened by disruption of the collagen type IV and that the cysts originate from the glomeruli or distal tubules because the collagen type IV α3, α4 and α5 chains are expressed only in these membranes [30, 37, 38].

The present study provides genetic, clinical and radiologic information of a cohort of 157 individuals from Spanish hospitals with heterozygous P/LP variants in *COL4A3/COL4A4* genes.

In the general population, the incidence of KC seems to increase with age, and this also held true in the present cohort [1–3, 39–42]. However, the prevalence of KC in the present cohort was higher than that in the age-matched general population studied by Mensel *et al.* [3] even though Mensel's cohort was assessed by MRI, where a higher sensitivity is expected. This finding supports the hypothesis that these individuals have a greater predisposition to develop bilateral KC. In the general population KC are more common in males; this was also true in the present cohort, although it did not reach statistical significance.

It is also known that patients with CKD are more prone to have KC and this correlates with the decline in eGFR [9, 16, 40, 43]. In the present cohort, worse eGFR was also associated with the presence of KC. However, even very mildly affected individuals showed an increased prevalence of KC. One study in healthy individuals in the general population found creatinine to be a risk factor for the development of KC, with serum creatinine 1.5 mg/dL (eGFR <60 mL/min/1.73 m^2^) as a cut-off point [9]. However, we have not been able to identify studies evaluating the presence of KC for each CKD category, although it is well known that ACKD occurs in advanced CKD categories (G4 and G5) and individuals on KRT. Our study shows that the presence of KC increases along with eGFR decline in individuals with heterozygous P/LP variants in the *COL4A3/COL4A4* genes. However, unlike what has been published in a smaller cohort [29], we do not observe an association with proteinuria in our cohort.

No genotype–phenotype association regarding presence of KC was identified in the present study. P/LP variants in other genes associated with ciliopathies that cause cystic kidney disease were ruled out, therefore collagen type IV variants are presumably the only genetic drivers of this feature. Cystic nephromegaly mimicking ADPKD has been reported in patients with *COL4A3/COL4A4* variants [30] but in the present series only 3.8% of patients had nephromegaly. In view of this very low percentage of patients with P/LP variants in the *COL4A3/COL4A4* genes with cystic nephromegaly, it seems advisable to exclude other inherited kidney diseases when cystic nephromegaly is present.

One limitation of our study is its inherent bias towards more severely affected individuals, which is a common characteristic of all clinical cohorts with P/LP variants in the *COL4A3/COL4A4*. This bias may be attributed to the underdiagnosis of individuals with a very mild or even nonexistent phenotype. Typically, individuals without kidney disease are not referred to nephrologists. However, the present cohort included individuals categorized as CKD-G1 and without proteinuria, most of them being relatives of the index case. Another limitation is the lack of evidence in the literature of the presence of KC in each CKD category, so we cannot make a direct comparison with other patients with CKD to determine whether this association is independent of eGFR.

In conclusion, we provide evidence that the prevalence of KC in individuals with P/LP variants in the *COL4A3*/*COL4A4* genes is higher than in the general population and is associated with aging and worsening eGFR (advancing CKD category). However, it has no clinical consequences, and no cyst-specific treatment is required. Larger studies of clinical features in individuals with P/LP variants in the *COL4A3/COL4A4* genes are needed to better understand this entity.

## Supplementary Material

gfae031_Supplemental_Files
