# Supplementary material for: Increased prevalence of kidney cysts in individuals carrying heterozygous COL4A3 or COL4A4 pathogenic variants
Source: Nephrol Dial Transplant. 2024 Feb 5;39(9):1442–8. doi: 10.1093/ndt/gfae031 (PMC11361806; doi:10.1093/ndt/gfae031)
Supplement: gfae031_Supplemental_Files [file gfae031_supplemental_files.zip › S1_Supplementary_Table 30_11_2023 word.docx]

| Family | Patient | Genetic  Study | Sex | *Gene* | DNA Variant | Protein variant | VariantClassif. | ACMG Criteria | Age | mH | eGFR | CKD | PCR mg/gCr | RK mm | LK  mm | RKC | LKC | Lithiasis | Nº RKC | Nº LKC | Ref. |
| --- | --- | --- | --- | --- | --- | --- | --- | --- | --- | --- | --- | --- | --- | --- | --- | --- | --- | --- | --- | --- | --- |
| ALP-0065 | 02-0001 | Panel | F | *COL4A3* | c.345del | p.(Pro116LeufsTer37) | P | PVS1, PM2_Supporting, PP1 | 80 | N | 83 | 2 | 62 | 94 | 105 | Y | N | N | 1 | 0 | Ref. 24 |
| ALP-0076 | 15-0288 | Panel | F | *COL4A3* | c.1345G>A | p.(Gly449Arg) | LP | PM1, PM2_Supporting, PP3_Moderate, PP1 | 19 | Y | 108 | 1 | 65 | 110 | 110 | N | N | N | 0 | 0 | Ref. 24 |
| ALP-0094 | 05-0130 | Panel | F | *COL4A3* | c.279+2T>C | p.? | LP | PVS1, PM2_Supporting | 35 | N | 84 | 2 | 4 | 100 | 100 | N | N | N | 0 | 0 | Ref. 24 |
| ALP-0094 | 09-0140 | Panel | M | *COL4A3* | c.279+2T>C | p.? | LP | PVS1, PM2_Supporting | 42 | Y | 76 | 2 | 20 | 110 | 110 | N | N | N | 0 | 0 | Ref. 24 |
| ALP-0115 | 15-0487 | Sanger | M | *COL4A3; COL4A4* | c.4826G>A; c.4531G>A (cis) | p.(Arg1609Gln); p.(Gly1451Arg) | LP; LP | PM2_Supporting, PP3_Moderate; PM1, PM2_Supporting, PP3_Moderate (in cis PM3, PP1) | 44 | Y | 91 | 1 | 36 | 110 | 110 | N | N | N | 0 | 0 | Ref. 24 |
| ALP-0144 | 08-0399 | Sanger | M | *COL4A3* | c.725G>A | p.(Gly242Glu) | LP | PM1, PM2_Supporting, PP3_Moderate, PP4 | 52 | Y | 50 | 3a | 230 | 130 | 122 | Y | Y | N | 3+ | 3+ | this study |
| ALP-0157 | 09-0200 | Panel | M | *COL4A3* | c.345del | p.(Pro116LeufsTer37) | P | PVS1, PM2_Supporting, PP1 | 60 | Y | 71 | 2 | 6 | 90 | 80 | N | N | N | 0 | 0 | Ref. 24 |
| ALP-0157 | 09-0201 | Panel | F | *COL4A3* | c.345del | p.(Pro116LeufsTer37) | P | PVS1, PM2_Supporting, PP1 | 50 | Y | 76 | 2 | 8 | 110 | 110 | N | N | N | 0 | 0 | Ref. 24 |
| ALP-0165 | 10-0046 | Panel | F | *COL4A3* | c.2954G>T | p.(Gly985Val) | LP | PM1, PM2_Supporting, PP3_Moderate, PP1 | 46 | Y | 83 | 2 | 4 | 110 | 110 | N | N | N | 0 | 0 | Ref. 24 |
| ALP-0165 | 10-0047 | Panel | F | *COL4A3* | c.2954G>T | p.(Gly985Val) | LP | PM1, PM2_Supporting, PP3_Moderate, PP1 | 20 | Y | 111 | 1 | 12 | 120 | 94 | N | N | N | 0 | 0 | Ref. 24 |
| ALP-0165 | 10-0419 | Sanger | F | *COL4A3* | c.2954G>T | p.(Gly985Val) | LP | PM1, PM2_Supporting, PP3_Moderate, PP1 | 46 | Y | 107 | 1 | 4 | 110 | 110 | N | N | N | 0 | 0 | Ref. 24 |
| ALP-0165 | 12-0244 | Panel | M | *COL4A3* | c.2954G>T | p.(Gly985Val) | LP | PM1, PM2_Supporting, PP3_Moderate, PP1 | 18 | Y | 110 | 1 | 4 | 113 | 110 | N | N | N | 0 | 0 | Ref. 24 |
| ALP-0166 | 10-0130 | Panel | M | *COL4A3* | c.3643C>T | p.(Arg1215Ter) | P | PVS1, PM2_Supporting, PP1 | 55 | N | 96 | 1 | 120 | 100 | 100 | Y | Y | N | 1 | 2 | Ref. 24 |
| ALP-0166 | 10-0131 | Sanger | F | *COL4A3* | c.3643C>T | p.(Arg1215Ter) | P | PVS1, PM2_Supporting, PP1 | 48 | N | 100 | 1 | 100 | 100 | 100 | N | Y | N | 0 | 1 | Ref. 24 |
| ALP-0166 | 11-0108 | Sanger | M | *COL4A3* | c.3643C>T | p.(Arg1215Ter) | P | PVS1, PM2_Supporting, PP1 | 37 | N | 107 | 1 | 80 | 100 | 100 | N | N | N | 0 | 0 | Ref. 24 |
| ALP-0174 | 11-0098 | Panel | F | *COL4A3* | c.2083G>A | p.(Gly695Arg) | LP | PM1, PM2_Supporting, PP1, PP3_Moderate | 66 | Y | 37 | 3b | 207 | 92 | 97 | Y | Y | N | 3+ | 3+ | Ref. 24 |
| ALP-0174 | 11-0100 | Panel | F | *COL4A3* | c.2083G>A | p.(Gly695Arg) | LP | PM1, PM2_Supporting, PP1, PP3_Moderate | 26 | Y | 93 | 1 | 4 | 110 | 110 | N | N | N | 0 | 0 | Ref. 24 |
| ALP-0188 | 17-0283 | Panel | M | *COL4A3* | c.1918G>A | p.(Gly640Arg) | LP | PM1, PM2_Supporting, PP3_Moderate, PP4 | 63 | Y | 33 | 3b | 80 | 110 | 110 | Y | N | N | 2 | 0 | Ref. 24 |
| ALP-0195 | 12-0281 | Panel | F | *COL4A4* | c.2628_2654dup | p.(Arg877_Gly885dup) | LP | PM2_Supporting, PM4, PP1_Moderate, PS4_Moderate | 60 | Y | 37 | 3b | 1800 | 90 | 90 | Y | Y | N | 3+ | 3+ | Ref. 24 |
| ALP-0195 | 12-0282 | Panel | F | *COL4A4* | c.2628_2654dup | p.(Arg877_Gly885dup) | LP | PM2_Supporting, PM4, PP1_Moderate, PS4_Moderate | 55 | Y | 101 | 1 | 400 | 101 | 106 | N | N | N | 0 | 0 | Ref. 24 |
| ALP-0195 | 12-0283 | Panel | F | *COL4A4* | c.2628_2654dup | p.(Arg877_Gly885dup) | LP | PM2_Supporting, PM4, PP1_Moderate, PS4_Moderate | 46 | Y | 76 | 2 | 144 | 100 | 100 | N | N | N | 0 | 0 | this study |
| ALP-0200 | 13-0230 | Panel | F | *COL4A3* | c.4253G>A | p.(Gly1487Glu) | LP | PM1, PM2_Supporting, PP3_Moderate, PP1 | 57 | Y | 35 | 3b | 1792 | 109 | 106 | Y | Y | N | 3+ | 3+ | Ref. 24 |
| ALP-0200 | 13-0232 | Panel | M | *COL4A3* | c.4253G>A | p.(Gly1487Glu) | LP | PM1, PM2_Supporting, PP3_Moderate, PP1 | 19 | Y | 98 | 1 | 70 | 110 | 110 | N | N | N | 0 | 0 | Ref. 24 |
| ALP-0200 | 13-0285 | Panel | F | *COL4A3* | c.4253G>A | p.(Gly1487Glu) | LP | PM1, PM2_Supporting, PP3_Moderate, PP1 | 47 | Y | 26 | 4 | 3000 | 89 | 83 | Y | Y | N | 3+ | 3+ | Ref. 24 |
| ALP-0200 | 13-0286 | Panel | F | *COL4A3* | c.4253G>A | p.(Gly1487Glu) | LP | PM1, PM2_Supporting, PP3_Moderate, PP1 | 20 | Y | 112 | 1 | 7 | 110 | 110 | N | N | N | 0 | 0 | Ref. 24 |
| ALP-0200 | 15-0550 | Sanger | F | *COL4A3* | c.4253G>A | p.(Gly1487Glu) | LP | PM1, PM2_Supporting, PP3_Moderate, PP1 | 39 | Y | 82 | 2 | 110 | 110 | 110 | N | N | N | 0 | 0 | Ref. 24 |
| ALP-0214 | 14-0216 | Panel | M | *COL4A4* | c.[2320G>C;4394G>A] cis | p.[Gly774Arg; Gly1465Asp] cis | LP | PM1, PM2_Supporting, PP3_Moderate, PP1, PS4 | 57 | Y | 14 | 5 | 7000 | 95 | 95 | Y | Y | N | 3+ | 3+ | Ref. 24 |
| ALP-0214 | 14-0218 | Panel | M | *COL4A4* | c.[2320G>C;4394G>A] cis | p.[Gly774Arg; Gly1465Asp] cis | LP | PM1, PM2_Supporting, PP3_Moderate, PP1, PS4 | 42 | Y | 78 | 2 | 5 | 100 | 100 | N | N | N | 0 | 0 | Ref. 24 |
| ALP-0223 | 14-0394 | Panel | F | *COL4A4* | c.1143_1144del | p.(Asp383CysfsTer46) | P | PVS1, PM2_Supporting, PP1 | 35 | Y | 101 | 1 | 6 | 110 | 110 | N | Y | Y | 0 | 1 | Ref. 24 |
| ALP-0223 | 15-0742 | Panel | F | *COL4A4* | c.1143_1144del | p.(Asp383CysfsTer46) | P | PVS1, PM2_Supporting, PP1 | 38 | Y | 86 | 2 | 3 | 105 | 104 | N | N | N | 0 | 0 | Ref. 24 |
| ALP-0226 | 14-0470 | Panel | F | *COL4A4* | c.735+2T>C | p.? | P | PVS1, PM2_Supporting, PP1 | 41 | Y | 81 | 2 | 275 | 100 | 100 | N | N | N | 0 | 0 | Ref. 24 |
| ALP-0231 | 10-0407 | Panel | F | *COL4A4* | c.3559G>A | p.(Gly1187Arg) | LP | PM1, PM2_Supporting, PP3_Moderate, PP1 | 28 | Y | 138 | 1 | 5 | 110 | 110 | N | N | N | 0 | 0 | Ref. 24 |
| ALP-0232 | 14-0542 | Panel | F | *COL4A4* | c.4334-23A>G | p.? | LP | PM2_Supporting, PM3, PP1, PS4 | 58 | Y | 49 | 3a | 290 | 91 | 99 | Y | Y | N | 3+ | 3+ | this study |
| ALP-0232 | 16-0550 | Panel | F | *COL4A4* | c.4334-23A>G | p.? | LP | PM2_Supporting, PM3, PP1, PS4 | 35 | Y | 114 | 1 | 48 | 100 | 100 | N | N | N | 0 | 0 | Ref. 24 |
| ALP-0235 | 22-0424 | Panel | M | *COL4A4* | c.2628_2654dup | p.(Arg877_Gly885dup) | LP | PM2_Supporting, PM4, PP1_Moderate, PS4_Moderate | 41 | Y | 67 | 2 | 627 | 99 | 112 | N | N | N | 0 | 0 | this study |
| ALP-0235 | 22-0434 | Panel | F | *COL4A4* | c.2628_2654dup | p.(Arg877_Gly885dup) | LP | PM2_Supporting, PM4, PP1_Moderate, PS4_Moderate | 41 | Y | 107 | 1 | 21 | 111 | 114 | N | N | N | 0 | 0 | this study |
| ALP-0235 | 22-0451 | Sanger | F | *COL4A4* | c.2628_2654dup | p.(Arg877_Gly885dup) | LP | PM2_Supporting, PM4, PP1_Moderate, PS4_Moderate | 37 | Y | 111 | 1 | 19 | 100 | 100 | N | N | N | 0 | 0 | this study |
| ALP-0238 | 15-0030 | Panel | F | *COL4A4* | c.735+2T>C | p.? | P | PVS1, PM2_Supporting, PP1 | 54 | Y | 31 | 3b | 2700 | 100 | 100 | N | N | N | 0 | 0 | Ref. 24 |
| ALP-0238 | 15-0031 | Panel | M | *COL4A4* | c.735+2T>C | p.? | P | PVS1, PM2_Supporting, PP1 | 48 | Y | 58 | 3a | 94 | 100 | 100 | Y | Y | N | 3+ | 3+ | Ref. 24 |
| ALP-0238 | 15-0032 | Panel | F | *COL4A4* | c.735+2T>C | p.? | P | PVS1, PM2_Supporting, PP1 | 44 | Y | 30 | 3b | 328 | 95 | 100 | Y | Y | N | 1 | 1 | Ref. 24 |
| ALP-0246 | 12-0417 | Panel | F | *COL4A4* | c.2219dup | p.(Val741CysfsTer47) | P | PVS1, PM2_Supporting, PS4_Supporting | 53 | Y | 58 | 3a | 4233 | 100 | 98 | N | N | N | 0 | 0 | Ref. 24 |
| ALP-0252 | 15-0527 | Panel | F | *COL4A3; COL4A4* | c.2342_2350del; c.5045G>A (cis) | p.(Thr781_Gly783del); p.(Arg1682Gln) | LP; LP | PM1, PM2_Supporting, PM4, PP1; PM2_Supporting, PP3_Moderate, PM5_Supporting, PS4_Moderate | 48 | Y | 98 | 1 | 19 | 99 | 110 | N | N | N | 0 | 0 | Ref. 24 |
| ALP-0252 | 16-0265 | Panel | M | *COL4A3; COL4A4* | c.2342_2350del; c.5045G>A (cis) | p.(Thr781_Gly783del); p.(Arg1682Gln) | LP; LP | PM1, PM2_Supporting, PM4, PP1; PM2_Supporting, PP3_Moderate, PM5_Supporting, PS4_Moderate | 50 | Y | 25 | 4 | 105 | 110 | 110 | N | N | N | 0 | 0 | Ref. 24 |
| ALP-0252 | 18-0006 | Sanger | F | *COL4A3; COL4A4* | c.2342_2350del; c.5045G>A (cis) | p.(Thr781_Gly783del); p.(Arg1682Gln) | LP; LP | PM1, PM2_Supporting, PM4, PP1; PM2_Supporting, PP3_Moderate, PM5_Supporting, PS4_Moderate | 20 | Y | 107 | 1 | 2 | 100 | 100 | N | N | N | 0 | 0 | this study |
| ALP-0253 | 07-0120 | Panel | M | *COL4A4* | c.114+1G>C | p.? | P | PVS1, PM2_Supporting, PP1 | 57 | Y | 15 | 5 | 677 | 98 | 80 | N | N | N | 0 | 0 | this study |
| ALP-0256 | 15-0567 | Panel | F | *COL4A3* | c.1391G>T | p.(Gly464Val) | P | PM1, PM2_Supporting, PP3_Moderate, PM5_Supporting, PS4 | 58 | Y | 70 | 2 | 5 | 110 | 110 | Y | Y | N | 3+ | 3+ | Ref. 24 |
| ALP-0268 | 09-0258 | Panel | F | *COL4A4* | c.1460G>T | p.(Gly487Val) | LP | PM1, PM2_Supporting, PP3_Moderate, PP1 | 67 | Y | 38 | 3b | 19 | 75 | 97 | N | N | N | 0 | 0 | Ref. 24 |
| ALP-0270 | 16-0109 | Panel | M | *COL4A3* | c.2954G>T | p.(Gly985Val) | LP | PM1, PM2_Supporting, PP3_Moderate, PP1 | 58 | Y | 29 | 4 | 448 | 98 | 105 | N | N | N | 0 | 0 | Ref. 24 |
| ALP-0273 | 07-0015 | Panel | F | *COL4A3* | c.3472G>C | p.(Gly1158Arg) | LP | PM1, PM2_Supporting, PP3_Moderate, PP1 | 35 | Y | 114 | 1 | 6 | 110 | 110 | N | N | N | 0 | 0 | Ref. 24 |
| ALP-0273 | 07-0440 | Panel | M | *COL4A3* | c.3472G>C | p.(Gly1158Arg) | LP | PM1, PM2_Supporting, PP3_Moderate, PP1 | 37 | Y | 101 | 1 | 207 | 132 | 132 | N | N | N | 0 | 0 | Ref. 24 |
| ALP-0275 | 16-0154 | Panel | M | *COL4A4* | c.3688G>T | p.(Gly1230Cys) | LP | PM1, PM2_Supporting, PP3_Moderate, PP1 | 67 | Y | 81 | 2 | 12 | 100 | 100 | N | Y | N | 0 | 1 | Ref. 24 |
| ALP-0280 | 16-0320 | Panel | M | *COL4A4* | c.3205G>C | p.(Gly1069Arg) | LP | PM1, PM2_Supporting, PP3_Moderate, PP1 | 25 | Y | 87 | 2 | 3160 | 117 | 127 | N | N | N | 0 | 0 | Ref. 24 |
| ALP-0280 | 16-0321 | Panel | M | *COL4A4* | c.3205G>C | p.(Gly1069Arg) | LP | PM1, PM2_Supporting, PP3_Moderate, PP1 | 32 | Y | 33 | 3b | 3600 | 91 | 92 | N | N | N | 0 | 0 | Ref. 24 |
| ALP-0281 | 16-0133 | Panel | M | *COL4A4* | c.4508del | p.(His1503ProfsTer49) | P | PVS1, PM2_Supporting, PP1 | 35 | Y | 37 | 3b | 3600 | 115 | 70 | N | N | N | 0 | 0 | Ref. 24 |
| ALP-0281 | 17-0882 | Panel | M | *COL4A4* | c.4508del | p.(His1503ProfsTer49) | P | PVS1, PM2_Supporting, PP1 | 42 | Y | 74 | 2 | 3000 | 100 | 100 | N | N | N | 0 | 0 | Ref. 24 |
| ALP-0281 | 17-0883 | Panel | M | *COL4A4* | c.4508del | p.(His1503ProfsTer49) | P | PVS1, PM2_Supporting, PP1 | 47 | Y | 42 | 3b | 1500 | 100 | 100 | Y | Y | N | 3+ | 3+ | Ref. 24 |
| ALP-0284 | 16-0416 | Panel | M | *COL4A4* | c.1323_1340del | p.(Pro444_Leu449del) | LP | PM1, PM2_Supporting, PM4, PP1 | 39 | Y | 91 | 1 | 1530 | 100 | 100 | Y | Y | N | 3+ | 3+ | Ref. 24 |
| ALP-0291 | 17-0350 | Sanger | F | *COL4A3* | c.2126G>A | p.(Gly709Glu) | LP | PM1, PM2_Supporting, PP1, PP3_Moderate, PS4_Supporting | 49 | Y | 121 | 1 | 2080 | 100 | 100 | N | N | N | 0 | 0 | Ref. 24 |
| ALP-0296 | 17-0042 | Panel | F | *COL4A3* | c.2126G>A | p.(Gly709Glu) | LP | PM1, PM2_Supporting, PP1, PP3_Moderate, PS4_Supporting | 41 | Y | 46 | 3a | 60 | 100 | 100 | N | N | N | 0 | 0 | Ref. 24 |
| ALP-0296 | 17-0043 | Panel | F | *COL4A3* | c.2126G>A | p.(Gly709Glu) | LP | PM1, PM2_Supporting, PP1, PP3_Moderate, PS4_Supporting | 36 | Y | 113 | 1 | 4 | 113 | 113 | N | N | N | 0 | 0 | Ref. 24 |
| ALP-0296 | 17-0044 | Panel | F | *COL4A3* | c.2126G>A | p.(Gly709Glu) | LP | PM1, PM2_Supporting, PP1, PP3_Moderate, PS4_Supporting | 35 | Y | 118 | 1 | 12 | 100 | 100 | Y | Y | N | 3+ | 3+ | Ref. 24 |
| ALP-0301 | 17-0108 | Panel | F | *COL4A3* | c.3133G>C | p.(Gly1045Arg) | LP | PM1, PM2_Supporting, PP3_Moderate, PP1, PP4 | 63 | Y | 41 | 3b | 503 | 105 | 105 | Y | N | N | 2 | 0 | Ref. 24 |
| ALP-0301 | 17-0513 | Panel | M | *COL4A3* | c.3133G>C | p.(Gly1045Arg) | LP | PM1, PM2_Supporting, PP3_Moderate, PP1, PP4 | 40 | Y | 87 | 2 | 10 | 108 | 102 | N | Y | N | 0 | 1 | Ref. 24 |
| ALP-0302 | 17-0129 | Panel | F | *COL4A4* | c.4334-23A>G | p.? | LP | PM2_Supporting, PM3, PP1, PS4 | 75 | Y | 42 | 3b | 89 | 100 | 100 | Y | N | N | 2 | 0 | Ref. 24 |
| ALP-0304 | 17-0142 | Panel | F | *COL4A3* | c.583G>A | p.(Gly195Ser) | LP | PM1, PM2_Supporting, PP3_Moderate, PP4 | 56 | Y | 21 | 4 | 4400 | 80 | 80 | N | N | N | 0 | 0 | Ref. 24 |
| ALP-0308 | 17-0159 | Panel | F | *COL4A3* | c.3499G>A | p.(Gly1167Arg) | LP | PM1, PM2_Supporting, PP3_Moderate, PP1, PS4_Moderate | 62 | Y | 38 | 3b | 42 | 82 | 85 | Y | N | N | 1 | 0 | Ref. 24 |
| ALP-0308 | 17-0160 | Panel | F | *COL4A3* | c.3499G>A | p.(Gly1167Arg) | LP | PM1, PM2_Supporting, PP3_Moderate, PP1, PS4_Moderate | 32 | Y | 110 | 1 | 9 | 100 | 100 | N | N | N | 0 | 0 | Ref. 24 |
| ALP-0310 | 10-0136 | Panel | M | *COL4A4* | c.3991G>T | p.(Gly1331Ter) | LP | PVS1, PM2_Supporting | 49 | Y | 77 | 2 | 1 | 110 | 110 | N | N | N | 0 | 0 | Ref. 24 |
| ALP-0313 | 17-0245 | Panel | M | *COL4A3* | c.2074G>A | p.(Gly692Ser) | LP | PM1, PM2_Supporting, PP3_Moderate, PP4 | 35 | Y | 37 | 3b | 560 | 110 | 110 | Y | Y | N | 1 | 1 | Ref. 24 |
| ALP-0329 | 17-0455 | Panel | M | *COL4A4* | c.2242G>A | p.(Gly748Ser) | LP | PM1, PM2_Supporting, PP3_Moderate, PP4 | 50 | Y | 99 | 1 | 8 | 100 | 100 | N | Y | N | 0 | 1 | Ref. 24 |
| ALP-0329 | 17-0456 | Panel | M | *COL4A4* | c.2242G>A | p.(Gly748Ser) | LP | PM1, PM2_Supporting, PP3_Moderate, PP4 | 53 | Y | 95 | 1 | 75 | 100 | 100 | N | N | N | 0 | 0 | Ref. 24 |
| ALP-0334 | 17-0498 | Panel | F | *COL4A3* | c.898G>A | p.(Gly300Arg) | LP | PM1, PM2_Supporting, PP3_Moderate, PP1, PP4 | 55 | Y | 49 | 3a | 2700 | 100 | 100 | Y | Y | N | 1 | 1 | Ref. 24 |
| ALP-0334 | 17-0499 | Panel | F | *COL4A3* | c.898G>A | p.(Gly300Arg) | LP | PM1, PM2_Supporting, PP3_Moderate, PP1, PP4 | 51 | Y | 38 | 3b | 570 | 85 | 104 | Y | Y | N | 3+ | 3+ | Ref. 24 |
| ALP-0334 | 17-0500 | Panel | F | *COL4A3* | c.898G>A | p.(Gly300Arg) | LP | PM1, PM2_Supporting, PP3_Moderate, PP1, PP4 | 37 | Y | 111 | 1 | 12 | 100 | 100 | N | N | N | 0 | 0 | Ref. 24 |
| ALP-0340 | 17-0627 | Panel | M | *COL4A4* | c.1952G>T | p.(Gly651Val) | LP | PM1, PM2_Supporting, PP3_Moderate, PS4_Moderate, PP1 | 38 | Y | 115 | 1 | 841 | 97 | 105 | N | N | N | 0 | 0 | Ref. 24 |
| ALP-0342 | 18-0144 | Panel | F | *COL4A3* | c.546+1del | p.(Gly183ValfsTer40) | LP | PVS1, PM2_Supporting | 46 | Y | 37 | 3b | 1344 | 106 | 107 | Y | Y | N | 2 | 2 | this study |
| ALP-0349 | 14-0309 | Sanger | M | *COL4A4* | c.755G>T | p.(Gly252Val) | LP | PM1, PM2_Supporting, PP3_Moderate, PP4 | 50 | Y | 48 | 3a | 34 | 107 | 111 | Y | N | N | 2 | 0 | Ref. 24 |
| ALP-0349 | 17-0547 | Panel | F | *COL4A4* | c.755G>T | p.(Gly252Val) | LP | PM1, PM2_Supporting, PP3_Moderate, PP4 | 37 | Y | 58 | 3a | 62 | 120 | 120 | N | Y | Y | 0 | 1 | Ref. 24 |
| ALP-0351 | 17-0764 | Sanger | M | *COL4A3* | c.1096G>A | p.(Gly366Arg) | LP | PM1, PM2_Supporting, PP3_Moderate, PS4_Moderate | 49 | Y | 54 | 3a | 4 | 100 | 100 | N | Y | N | 0 | 1 | Ref. 24 |
| ALP-0372 | 18-0012 | Panel | F | *COL4A4* | c.4764T>G | p.(Cys1588Trp) | LP | PM1, PM2_Supporting, PP3_Moderate, PS4_Moderate | 42 | Y | 88 | 2 | 619 | 114 | 99 | N | N | N | 0 | 0 | this study |
| ALP-0384 | 18-0202 | Panel | M | *COL4A4* | c.2628_2654dup | p.(Arg877_Gly885dup) | LP | PM2_Supporting, PM4, PP1_Moderate, PS4_Moderate | 82 | Y | 16 | 4 | 1700 | 90 | 90 | N | Y | N | 0 | 1 | Ref. 24 |
| ALP-0393 | 18-0320 | Panel | F | *COL4A4* | c.[2320G>C;4394G>A] cis | p.[Gly774Arg; Gly1465Asp] cis | LP | PM1, PM2_Supporting, PP3_Moderate, PP1, PS4 | 61 | Y | 68 | 2 | 34 | 114 | 110 | Y | Y | N | 2 | 1 | this study |
| ALP-0393 | 18-0321 | Panel | F | *COL4A4* | c.[2320G>C;4394G>A] cis | p.[Gly774Arg; Gly1465Asp] cis | LP | PM1, PM2_Supporting, PP3_Moderate, PP1, PS4 | 57 | Y | 107 | 1 | 25 | 124 | 114 | Y | Y | N | 3+ | 3+ | this study |
| ALP-0396 | 22-0441 | Panel | M | *COL4A3* | c.4981C>T | p.(Arg1661Cys) | LP | PP3_Moderate, PM3, PS4_Moderate | 61 | Y | 82 | 2 | 5 | 102 | 104 | Y | N | N | 1 | 0 | this study |
| ALP-0396 | 67FJD7164 | Sanger | M | *COL4A3* | c.4981C>T | p.(Arg1661Cys) | LP | PP3_Moderate, PM3, PS4_Moderate | 44 | Y | 65 | 2 | 10 | 107 | 110 | N | Y | N | 0 | 1 | this study |
| ALP-0423 | 22-0440 | MLPA | F | *COL4A3; COL4A4* | EX1del; (EX1-EX48)del | p.?; p.? | LP; LP | PVS1, PM2_Supporting; PVS1, PM2_Supporting | 52 | Y | 59 | 3a | 190 | 94 | 87 | N | N | N | 0 | 0 | this study |
| ALP-0457 | 19-0841 | Panel | F | *COL4A4* | c.3488G>A | p.(Gly1163Asp) | LP | PM1, PM2_Supporting, PP3_Moderate, PP4 | 62 | Y | 94 | 1 | 1761 | 104 | 101 | N | N | N | 0 | 0 | this study |
| ALP-0463 | 12-0222 | Panel | M | *COL4A3* | EX22del | p.? | P | PVS1, PM2_Supporting, PP1 | 79 | Y | 42 | 3b | 4 | 122 | 110 | Y | Y | N | 3+ | 3+ | this study |
| ALP-0463 | 19-0916 | MLPA | F | *COL4A3* | EX22del | p.? | P | PVS1, PM2_Supporting, PP1 | 39 | Y | 112 | 1 | 7 | 124 | 124 | N | N | N | 0 | 0 | this study |
| ALP-0463 | 20-0517 | Panel | M | *COL4A3* | EX22del | p.? | P | PVS1, PM2_Supporting, PP1 | 46 | Y | 105 | 1 | 15 | 133 | 125 | Y | Y | N | 1 | 1 | this study |
| ALP-0481 | 19-0620 | Panel | F | *COL4A4* | c.3717_3725del | p.(Pro1241_Pro1243del) | LP | PM1, PM2_Supporting, PM4, PS4_Supporting | 51 | Y | 64 | 2 | 144 | 90 | 120 | Y | Y | N | 1 | 3+ | this study |
| ALP-0485 | 19-0694 | Sanger | M | *COL4A4* | c.320G>A | p.(Gly107Glu) | LP | PM1, PM2_Supporting, PP3_Moderate, PP4 | 22 | Y | 118 | 1 | 18 | 117 | 113 | N | N | Y | 0 | 0 | this study |
| ALP-0503 | 20-0003 | Panel | F | *COL4A4* | c.2617G>A | p.(Gly873Arg) | LP | PM1, PM2_Supporting, PP3_Moderate, PP4, PP1 | 38 | Y | 56 | 3a | 284 | 139 | 145 | Y | Y | N | 3+ | 3+ | this study |
| ALP-0503 | 20-0427 | Sanger | M | *COL4A4* | c.2617G>A | p.(Gly873Arg) | LP | PM1, PM2_Supporting, PP3_Moderate, PP4, PP1 | 66 | Y | 90 | 1 | 267 | 112 | 121 | Y | Y | N | 3+ | 3+ | this study |
| ALP-0504 | 20-0006 | Panel | F | *COL4A4* | c.1506del | p.(Gly503AlafsTer150) | LP | PVS1, PM2_Supporting | 66 | Y | 27 | 4 | 1787 | 94 | 113 | Y | Y | N | 3+ | 3+ | this study |
| ALP-0513 | 21-0089 | Sanger | F | *COL4A3* | c.1901G>A | p.(Gly634Glu) | LP | PM1, PM2_Supporting, PP3_Moderate, PP4, PP1 | 78 | N | 21 | 4 | 131 | 80 | 80 | Y | Y | N | 3+ | 3+ | this study |
| ALP-0513 | 20-0074 | Panel | M | *COL4A3; COL4A4* | c.1901G>A; c.1607G>A | p.(Gly634Glu); p.(Gly536Glu) | LP; LP | PM1, PM2_Supporting, PP3_Moderate, PP4, PP1; PM1, PM2_Supporting, PP3_Moderate, PP4 | 54 | Y | 28 | 4 | 977 | 97 | 97 | Y | Y | N | 3+ | 3+ | this study |
| ALP-0521 | 17-0361 | Panel | M | *COL4A4* | c.2628_2654dup | p.(Arg877_Gly885dup) | LP | PM2_Supporting, PM4, PP1_Moderate, PS4_Moderate | 59 | Y | 46 | 3a | 3 | 110 | 100 | Y | N | N | 1 | 0 | this study |
| ALP-0531 | 20-0262 | Panel | F | *COL4A4* | c.2429G>A | p.(Gly810Asp) | LP | PM1, PM2_Supporting, PP1, PP3_Moderate, PS4_Moderate | 37 | Y | 94 | 1 | 737 | 100 | 105 | Y | Y | N | 1 | 1 | this study |
| ALP-0551 | 20-0416 | Panel | M | *COL4A4* | c.658G>A | p.(Gly220Arg) | LP | PM1, PM2_Supporting, PP3_Moderate, PP4 | 44 | Y | 101 | 1 | 424 | 113 | 111 | Y | Y | N | 3+ | 3+ | this study |
| ALP-0555 | 20-0479 | Panel | M | *COL4A3* | c.2126G>A | p.(Gly709Glu) | LP | PM1, PM2_Supporting, PP1, PP3_Moderate, PS4_Supporting | 68 | N | 39 | 3b | 825 | 95 | 105 | Y | Y | N | 3+ | 3+ | this study |
| ALP-0575 | 20-0595 | Panel | F | *COL4A4* | c.1135G>A | p.(Gly379Arg) | LP | PM1, PM2_Supporting, PP3_Moderate, PP4 | 49 | Y | 90 | 1 | 20 | 120 | 112 | Y | N | N | 1 | 0 | this study |
| ALP-0586 | 21-0463 | Sanger | M | *COL4A4* | c.2628_2654dup | p.(Arg877_Gly885dup) | LP | PM2_Supporting, PM4, PP1_Moderate, PS4_Moderate | 22 | Y | 125 | 1 | 40 | 131 | 118 | N | Y | NA | 0 | 1 | this study |
| ALP-0592 | 21-0173 | Panel | F | *COL4A3* | c.2321G>T | p.(Gly774Val) | LP | PM1, PM2_Supporting, PP3_Moderate, PP1 | 54 | Y | 99 | 1 | 104 | 116 | 130 | N | N | NA | 0 | 0 | this study |
| ALP-0596 | 21-0383 | Sanger | M | *COL4A4* | c.[2320G>C;4394G>A] cis | p.[Gly774Arg; Gly1465Asp] cis | LP | PM1, PM2_Supporting, PP3_Moderate, PP1, PS4 | 31 | Y | 116 | 1 | 33 | 111 | 108 | Y | Y | NA | 1 | 1 | this study |
| ALP-0602 | FP-888 | Sanger | F | *COL4A4* | c.997_1014del | p.(Asp333_Gly338del) | LP | PM1, PM2_Supporting, PM4, PP4 | 54 | Y | 79 | 2 | 256 | 114 | 115 | N | N | N | 0 | 0 | this study |
| ALP-0606 | 21-0264 | Panel | F | *COL4A4* | c.2437G>T | p.(Gly813Ter) | LP | PVS1, PM2_Supporting | 51 | Y | 101 | 1 | 718 | 119 | 129 | N | Y | N | 0 | 1 | this study |
| ALP-0622 | 21-0549 | Panel | M | *COL4A4* | c.5045G>A | p.(Arg1682Gln) | LP | PM2_Supporting, PP3_Moderate, PM5_Supporting, PS4_Moderate | 58 | Y | 57 | 3a | 30 | 113 | 113 | Y | Y | NA | 3+ | 3+ | this study |
| ALP-0628 | 21-0494 | Panel | F | *COL4A3* | (EX10-EX11)del | p.? | LP | PVS1, PM2_Supporting | 54 | Y | 89 | 2 | 420 | 100 | 100 | Y | Y | NA | 0 | 0 | this study |
| ALP-0638 | 21-0557 | Panel | M | *COL4A4* | c.[2320G>C;4394G>A] cis | p.[Gly774Arg; Gly1465Asp] cis | LP | PM1, PM2_Supporting, PP3_Moderate, PP1, PS4 | 72 | Y | 56 | 3a | 331 | 95 | 95 | Y | Y | NA | 3+ | 3+ | this study |
| ALP-0647 | 21-0632 | Panel | F | *COL4A4* | c.2429G>A | p.(Gly810Asp) | LP | PM1, PM2_Supporting, PP1, PP3_Moderate, PS4_Moderate | 32 | Y | 124 | 1 | 94 | 100 | 100 | N | N | NA | 0 | 0 | this study |
| ALP-0647 | 21-0633 | Panel | M | *COL4A4* | c.2429G>A | p.(Gly810Asp) | LP | PM1, PM2_Supporting, PP1, PP3_Moderate, PS4_Moderate | 56 | Y | 60 | 2 | 30 | 120 | 115 | N | N | NA | 0 | 0 | this study |
| ALP-0676 | 22-0287 | Panel | F | *COL4A4* | c.114+1G>C | p.? | P | PVS1, PM2_Supporting, PP1 | 72 | Y | 66 | 2 | 106 | 68 | 89 | N | Y | NA | 0 | 1 | this study |
| ALP-0683 | 22-0332 | MLPA | M | *COL4A3; COL4A4* | (EX1-EX51)del; (EX1-EX10)del | p.?; p.? | LP; LP | PVS1, PM2_Supporting; PVS1, PM2_Supporting | 71 | Y | 36 | 4 | 759 | 108 | 106 | Y | Y | NA | 3+ | 3+ | this study |
| ALP-0683 | 19-0725 | MLPA | F | *COL4A3; COL4A4* | (EX1-EX51)del; (EX1-EX10)del | p.?; p.? | LP; LP | PVS1, PM2_Supporting; PVS1, PM2_Supporting | 45 | Y | 72 | 2 | 88 | 99 | 100 | N | Y | N | 0 | 2 | this study |
| ALP-0694 | 22-0489 | Panel | F | *COL4A3* | c.1805_1828del | p.(Gly602_Pro609del) | LP | PM1, PM2_Supporting, PM4, PP1 | 56 | Y | 43 | 4 | 153 | 121 | 106 | Y | Y | NA | 3+ | 3+ | this study |
| ALP-0723 | 22-0426 | Panel | F | *COL4A3* | c.279+2T>C | p.? | LP | PVS1, PM2_Supporting | 68 | Y | 45 | 3a | 594 | 81 | 109 | Y | Y | N | 3+ | 3+ | this study |
| ALP-0729 | 22-0435 | Panel | F | *COL4A4* | (EX38-EX41)del | p.? | LP | PVS1, PM2_Supporting | 58 | Y | 94 | 1 | 3 | 92 | 90 | N | N | N | 0 | 0 | this study |
| ALP-0731 | 22-0437 | Panel | F | *COL4A3* | c.279+2T>C | p.? | LP | PVS1, PM2_Supporting | 47 | Y | 83 | 2 | 50 | 102 | 116 | Y | N | N | 1 | 0 | this study |
| ALP-0732 | 22-0438 | Panel | F | *COL4A4* | c.1334G>C | p.(Gly445Ala) | LP | PM1, PM2_Supporting, PP3_Moderate, PP4 | 54 | Y | 77 | 2 | 45 | 104 | 102 | N | N | N | 0 | 0 | this study |
| ALP-0732 | FJD-047 | Sanger | M | *COL4A4* | c.1334G>C | p.(Gly445Ala) | LP | PM1, PM2_Supporting, PP3_Moderate, PP4 | 49 | Y | 24 | 4 | 903 | 108 | 123 | N | N | N | 0 | 0 | this study |
| ALP-0737 | 22-0444 | Sanger | M | *COL4A4* | c.1030G>A | p.(Gly344Arg) | LP | PM1, PM2_Supporting, PP3_Moderate, PP4 | 41 | Y | 42 | 3b | 12 | 111 | 102 | N | N | N | 0 | 0 | this study |
| ALP-0738 | 22-0446 | Sanger | F | *COL4A3* | c.1219G>C | p.(Gly407Arg) | LP | PM1, PM2_Supporting, PP3_Moderate, PS4_Moderate | 39 | Y | 109 | 1 | 5 | 100 | 109 | N | N | N | 0 | 0 | this study |
| ALP-0739 | 22-0447 | MLPA | F | *COL4A3; COL4A4* | (EX1-EX52)del; (EX1-EX11)del | p.?; p.? | LP; LP | PVS1, PM2_Supporting; PVS1, PM2_Supporting | 38 | Y | 101 | 1 | 288 | 107 | 101 | N | N | N | 0 | 0 | this study |
| ALP-0740 | 22-0448 | Sanger | F | *COL4A3* | c.898G>A | p.(Gly300Arg) | LP | PM1, PM2_Supporting, PP3_Moderate, PP1, PP4 | 39 | Y | 108 | 1 | 30 | 102 | 112 | N | N | N | 0 | 0 | this study |
| ALP-0741 | 22-0449 | Sanger | M | *COL4A4* | c.3214+1G>A | p.? | LP | PVS1, PM2_Supporting | 34 | Y | 89 | 2 | 4 | 80 | 113 | N | N | N | 0 | 0 | this study |
| ALP-0742 | 22-0450 | Sanger | F | *COL4A4* | c.2628_2654dup | p.(Arg877_Gly885dup) | LP | PM2_Supporting, PM4, PP1_Moderate, PS4_Moderate | 51 | Y | 64 | 2 | 144 | 90 | 120 | Y | Y | N | 3+ | 3+ | this study |
| ALP-0748 | 22-0457 | Panel | F | *COL4A3* | c.2954G>T | p.(Gly985Val) | LP | PM1, PM2_Supporting, PP3_Moderate, PP1 | 28 | Y | 124 | 1 | 10 | 104 | 109 | N | Y | N | 0 | 1 | this study |
| ALP-0748 | FJD-009 | Sanger | F | *COL4A3* | c.2954G>T | p.(Gly985Val) | LP | PM1, PM2_Supporting, PP3_Moderate, PP1 | 28 | Y | 117 | 1 | 10 | 100 | 100 | Y | Y | N | 3+ | 3+ | this study |
| ALP-0763 | 23-0059 | Panel | M | *COL4A4* | c.1321_1369+3del | p.? | LP | PVS1, PM2_Supporting | 31 | Y | 53 | 3a | 34 | 101 | 108 | N | Y | NA | 0 | 1 | this study |
| ALP-0768 | 22-0650 | Panel | M | *COL4A4* | c.2219dup | p.(Val741CysfsTer47) | P | PVS1, PM2_Supporting, PS4_Supporting | 55 | Y | 80 | 2 | 64 | 100 | 100 | Y | Y | NA | 3+ | 3+ | this study |
| FC001 | C001 | Sanger | F | *COL4A3* | c.3499G>A | p.(Gly1167Arg) | LP | PM1, PM2_Supporting, PP3_Moderate, PP1, PS4_Moderate | 46 | Y | 14 | 5 | 1518 | 90 | 82 | Y | N | N | 3+ | 0 | this study |
| FC002 | C002 | Sanger | F | *COL4A4* | c.2429G>A | p.(Gly810Asp) | LP | PM1, PM2_Supporting, PP1, PP3_Moderate, PS4_Moderate | 66 | Y | 24 | 4 | 75 | 88 | 90 | Y | Y | N | 3+ | 3+ | this study |
| FC002 | C003 | Sanger | F | *COL4A4* | c.2429G>A | p.(Gly810Asp) | LP | PM1, PM2_Supporting, PP1, PP3_Moderate, PS4_Moderate | 34 | N | 89 | 2 | 302 | 95 | 105 | N | N | N | 0 | 0 | this study |
| FC002 | C004 | Sanger | M | *COL4A4* | c.2429G>A | p.(Gly810Asp) | LP | PM1, PM2_Supporting, PP1, PP3_Moderate, PS4_Moderate | 63 | Y | 8 | 5 | 5 | 120 | 129 | Y | Y | N | 3+ | 3+ | this study |
| FC002 | C005 | Sanger | M | *COL4A4* | c.2429G>A | p.(Gly810Asp) | LP | PM1, PM2_Supporting, PP1, PP3_Moderate, PS4_Moderate | 40 | N | 111 | 1 | 5 | 101 | 128 | N | N | N | 0 | 0 | this study |
| FC003 | C006 | Sanger | F | *COL4A4* | c.3933C>A | p.(Tyr1311Ter) | P | PVS1, PM2_Supporting, PP1 | 51 | Y | 102 | 1 | 200 | 100 | 100 | N | Y | N | 0 | 2 | this study |
| FC003 | C010 | Sanger | F | *COL4A4* | c.3933C>A | p.(Tyr1311Ter) | P | PVS1, PM2_Supporting, PP1 | 60 | Y | 18 | 4 | 257 | 80 | 80 | Y | Y | N | 3+ | 3+ | this study |
| FC003 | C013 | Sanger | M | *COL4A4* | c.3933C>A | p.(Tyr1311Ter) | P | PVS1, PM2_Supporting, PP1 | 27 | Y | 124 | 1 | 18 | 112 | 120 | N | N | N | 0 | 0 | this study |
| FC003 | C018 | Sanger | M | *COL4A4* | c.3933C>A | p.(Tyr1311Ter) | P | PVS1, PM2_Supporting, PP1 | 54 | Y | 84 | 2 | 76 | 105 | 118 | Y | Y | N | 3+ | 3+ | this study |
| FC003 | C020 | Sanger | F | *COL4A4* | c.3933C>A | p.(Tyr1311Ter) | P | PVS1, PM2_Supporting, PP1 | 43 | Y | 118 | 1 | 415 | 100 | 100 | Y | N | N | 2 | 0 | this study |
| FC004 | C007 | Sanger | F | *COL4A3* | c.2021-6_2023del | p.? | LP | PVS1, PM2_Supporting | 37 | Y | 123 | 1 | 25 | 100 | 100 | N | N | N | 0 | 0 | this study |
| FC005 | C008 | Sanger | M | *COL4A3* | c.1096G>A | p.(Gly366Arg) | LP | PM1, PM2_Supporting, PP3_Moderate, PS4_Moderate | 50 | Y | 74 | 2 | 5 | 100 | 100 | N | N | N | 0 | 0 | this study |
| FC006 | C009 | Sanger | F | *COL4A4* | c.2429G>A | p.(Gly810Asp) | LP | PM1, PM2_Supporting, PP1, PP3_Moderate, PS4_Moderate | 36 | Y | 12 | 5 | 5 | 93 | 78 | Y | Y | N | 3+ | 3+ | this study |
| FC007 | C011 | Sanger | F | *COL4A3* | c.2083G>A | p.(Gly695Arg) | LP | PM1, PM2_Supporting, PP1, PP3_Moderate | 31 | Y | 78 | 2 | 661 | 98 | 112 | Y | Y | N | 1 | 1 | this study |
| FC007 | C012 | Sanger | M | *COL4A3* | c.2083G>A | p.(Gly695Arg) | LP | PM1, PM2_Supporting, PP1, PP3_Moderate | 43 | N | 74 | 2 | 200 | 100 | 100 | N | Y | N | 0 | 1 | this study |
| FC010 | C017 | Sanger | F | *COL4A4* | c.2717G>T | p.(Gly906Val) | LP | PM1, PM2_Supporting, PP3_Moderate, PP4 | 54 | Y | 104 | 1 | 246 | 100 | 100 | N | Y | N | 0 | 1 | this study |
| FC011 | C019 | Sanger | F | *COL4A4* | c.1117G>A | p.(Gly373Arg) | LP | PM1, PM2_Supporting, PP3_Moderate, PP4 | 44 | N | 55 | 3a | 753 | 99 | 101 | N | N | N | 0 | 0 | this study |
| FC012 | C021 | Sanger | F | *COL4A4* | c.2762G>A | p.(Gly921Glu) | LP | PM1, PM2_Supporting, PP3_Moderate, PP4 | 64 | Y | 39 | 3b | 522 | 100 | 100 | Y | N | N | 3+ | 0 | this study |
| 12FJD  65719 | 12FJD  65719 | Sanger | M | *COL4A3* | c.4981C>T | p.(Arg1661Cys) | LP | PP3_Moderate, PM3, PS4_Moderate | 56 | Y | 31 | 3b | 763 | 94 | 96 | Y | Y | N | 3+ | 3+ | this study |
| 19FJD  19585 | FJD-052 | Sanger | F | *COL4A4* | c.2752G>A | p.(Gly918Arg) | LP | PM1, PM2_Supporting, PP1, PP3_Moderate, PP4 | 42 | Y | 109 | 1 | 21 | 112 | 117 | N | N | N | 0 | 0 | this study |
| 20FJD  88484 | FJD-055 | Sanger | M | *COL4A4* | c.755G>A | p.(Gly252Asp) | LP | PM1, PM2_Supporting, PP3_Moderate, PP4, PP1 | 61 | Y | 40 | 3b | 422 | 105 | 105 | N | N | N | 0 | 0 | this study |
| NFNF-0105 | 19-0047 | Panel | M | *COL4A4* | c.2908C>T | p.(Gln970Ter) | LP | PVS1, PM2_Supporting | 64 | Y | 47 | 3a | 4 | 110 | 116 | Y | Y | N | 3+ | 3+ | this study |
| NFNF-0117 | 12-0508 | Panel | M | *COL4A4* | c.2628_2654dup | p.(Arg877_Gly885dup) | LP | PM2_Supporting, PM4, PP1_Moderate, PS4_Moderate | 51 | Y | 38 | 3b | 761 | 170 | 150 | Y | Y | N | 3+ | 3+ | this study |
| NFNF-0167 | 23-0058 | Panel | F | *COL4A4* | c.801_802del | p.(Tyr268Ter) | LP | PVS1, PM2_Supporting | 66 | Y | 35 | 4 | 190 | 101 | 97 | Y | Y | NA | 3+ | 3+ | this study |
| NFNF-0421 | 17-0642 | Panel | M | *COL4A3* | c.1450G>T | p.(Gly484Arg) | LP | PM1, PM2_Supporting, PP3_Moderate, PM5_Supporting | 54 | N | 108 | 1 | 0 | 110 | 100 | Y | Y | N | 3+ | 3+ | this study |
| NFNF-0977 | 22-0640 | Panel | M | *COL4A4* | c.5045G>A | p.(Arg1682Gln) | LP | PM2_Supporting, PP3_Moderate, PM5_Supporting, PS4_Moderate | 65 | Y | 54 | 3a | 65 | 158 | 135 | Y | Y | NA | 3+ | 3+ | this study |
