# Supplementary material for: Increased prevalence of kidney cysts in individuals carrying heterozygous COL4A3 or COL4A4 pathogenic variants
Source: Nephrol Dial Transplant. 2024 Feb 5;39(9):1442–8. doi: 10.1093/ndt/gfae031 (PMC11361806; doi:10.1093/ndt/gfae031)
Supplement: gfae031_Supplemental_Files [file gfae031_supplemental_files.zip › S2_Supplementary_Table_85 genes related to kidney cysts_Bioinformatics Analysis.docx]

**Supplementary Table S2.** List of 85 genes related to kidney cysts contained in the kidney-disease gene panel.

| **Gene** | **OMIM ID** | **Description** |
| --- | --- | --- |
| *AHI1* | 608894 | Abelson helper integration site 1 |
| *ALMS1* | 606844 | ALMS1, centrosome and basal body associated protein |
| *ANKS6* | 615370 | Ankyrin repeat and sterile alpha motif domain containing 6 |
| *ALG8* | 608103 | Alpha-1,3-gluocosyltransferase |
| *ARL6* | 608845 | ADP ribosylation factor like GTPase 6 |
| *ATXN10* | 611150 | Ataxin 10 |
| *B9D1* | 614144 | B9 domain containing 1 |
| *B9D2* | 611951 | B9 protein domain 2 |
| *BBS1* | 209901 | Bardet-Biedl syndrome 1 |
| *BBS10* | 610148 | Bardet-Biedl syndrome 10 |
| *BBS12* | 610683 | Bardet-Biedl syndrome 12 |
| *BBS2* | 606151 | Bardet-Biedl syndrome 2 |
| *BBS4* | 600374 | Bardet-Biedl syndrome 4 |
| *BBS5* | 603650 | Bardet-Biedl syndrome 5 |
| *BBS7* | 607590 | Bardet-Biedl syndrome 7 |
| *BBS9* | 600374 | Bardet-Biedl syndrome 9 |
| *CC2D2A* | 612013 | Coiled-coil and C2 domain containing 2A |
| *CEP164* | 614848 | Centrosomal protein 164 |
| *CEP290* | 610142 | Centrosomal protein 290 |
| *CEP41* | 610523 | Centrosomal protein 41 |
| *COL4A1* | 120130 | Collagen type IV alpha 1 chain |
| *CSPP1* | 611654 | Centrosome and spindle pole associated protein 1 |
| *CYP24A1* | 126065 | Cytochrome P450, family 24, subfamily A, polypeptide 1 |
| *DYNC2H1* | 603297 | Dynein cytoplasmic 2 heavy chain 1 |
| *FAN1* | 613534 | FANCD2- and FANCI-associated nuclease 1 |
| *FLCN* | 607273 | Folliculin |
| *GLIS2* | 608539 | GLIS family zinc finger 2 |
| *HNF1B* | 189907 | HNF1 homeobox B |
| *HOGA1* | 613597 | 4-Hydroxy-2-oxoglutarate aldolase 1 |
| *IFT122* | 606045 | Intraflagellar transport 122 |
| *IFT140* | 614620 | Intraflagellar transport 140 |
| *IFT172* | 607386 | Intraflagellar transport 172 |
| *IFT43* | 614068 | Intraflagellar transport 43 |
| *INVS* | 243305 | Inversin |
| *IQCB1* | 609237 | IQ motif containing B1 |
| *JAG1* | 601920 | Jagged 1 |
| *KIF14* | 611279 | Kinesin family member 14 |
| *LZTFL1* | 606568 | Leucine zipper transcription factor like 1 |
| *MKKS* | 604896 | McKusick-Kaufman syndrome |
| *MKS1* | 609883 | Meckel syndrome, type 1 |
| *MUC1* | 158340 | Mucin 1, cell surface associated |
| *NEK1* | 604588 | NIMA-related kinase 1 |
| *NEK8* | 609799 | NIMA-related kinase 8 |
| *NOTCH2* | 600275 | Notch 2 |
| *NPHP1* | 607100 | Nephrocystin 1 |
| *NPHP3* | 608002 | Nephrocystin 3 |
| *NPHP4* | 607215 | Nephrocystin 4 |
| *OFD1* | 300170 | OFD1, centriole and centriolar satellite protein |
| *PDE6D* | 602676 | Phosphodiesterase 6D |
| *PDE6D* | 602676 | Phosphodiesterase 6D |
| *PMM2* | 601785 | Phosphomannomutase 2 |
| *PKD1* | 601313 | Polycystin 1 |
| *PKD2* | 173910 | Polycystin 2 |
| *PKHD1* | 606702 | Fibrocystin/polyductin |
| *POC1B* | 614783 | POC1 centriolar protein B |
| *PRKCSH* | 177060 | Protein kinase C substrate 80K-H |
| *REN* | 179820 | Renin |
| *RPGRIP1L* | 610937 | RPGRIP1-like |
| *SCLT1* | 611399 | Sodium channel and clathrin linker 1 |
| *SDCCAG8* | 613524 | Serologically defined colon cancer antigen 8 |
| *SEC63* | 608648 | SEC63 homolog, protein translocation regulator |
| *SLC34A3* | 609826 | Solute carrier family 34 member 3 |
| *SLC41A1* | 610801 | Solute carrier family 41 member 1 |
| *TCTN2* | 613846 | Tectonic family member 2 |
| *TFAP2A* | 107580 | Transcription factor AP-2 alpha |
| *TMEM138* | 614459 | Transmembrane protein 138 |
| *TMEM216* | 613277 | Transmembrane protein 216 |
| *TMEM231* | 614949 | Transmembrane protein 231 |
| *TMEM237* | 614423 | Transmembrane protein 237 |
| *TMEM67* | 609884 | Transmembrane protein 67 |
| *TRAF3IP1* | 607380 | TRAF3 interacting protein 1 |
| *TRIM32* | 602290 | Tripartite motif containing 32 |
| *TSC1* | 605284 | TSC complex subunit 1 |
| *TSC2* | 191092 | TSC complex subunit 2 |
| *TTC21B* | 612014 | Tetratricopeptide repeat domain 21B |
| *TTC8* | 608132 | Tetratricopeptide repeat domain 8 |
| *UMOD* | 191845 | Uromodulin |
| *VHL* | 608537 | Von Hippel-Lindau tumor suppressor |
| *WDPCP* | 613580 | WD repeat containing planar cell polarity effector |
| *WDR19* | 608151 | WD repeat domain 19 |
| *WDR34* | 613363 | WD repeat domain 34 |
| *WDR35* | 613602 | WD repeat domain 35 |
| *WDR60* | 615462 | WD repeat domain 60 |
| *XPNPEP3* | 613553 | X-prolyl aminopeptidase 3 |
| *ZNF423* | 604557 | Zinc finger protein 423 |

**Bioinformatics Analysis**

All the bioinformatics tools used in this study were run using default settings unless otherwise stated. Reads were aligned to the human reference genome hg19 using the Burrows-Wheeler Aligner [1]. Alignments were converted to BAM format and sorted using samtools [2]. Local realignment around potential insertions/deletions and SNP clusters and base-quality recalibration was performed using the GATK4 pipeline [3]. The resulting alignments were used as input for prediction tool GATK4 HaplotypeCaller [4]. The resulting variants were subsequently quality filtered using GATK VariantFiltration (parameters MQ < 0.0 || QUAL <25.0 || QD <0.0 || DP <5 || GQ <15). CoNVaDING (Copy Number Variation Detection In Next-generation sequencing Gene panels) was used to identify large indels and structural variants by read-depth analysis [5].

References:

1. Li H, Durbin R. Fast and accurate short read alignment with Burrows-Wheeler transform. Bioinformatics (Oxford, England) 2009; 25: 1754–1760.

2. Li H, Handsaker B, Wysoker A et al. The Sequence Alignment/Map format and SAMtools. Bioinformatics 2009; 25: 2078–2079.

3. McKenna A, Hanna M, Banks E et al. The genome analysis toolkit: A MapReduce framework for analyzing next-generation DNA sequencing data. Genome Research 2010; 20: 1297–1303.

4. Poplin R, Ruano-Rubio V, DePristo MA et al. Scaling accurate genetic variant discovery to tens of thousands of samples. bioRxiv 2017; : 201178.

5. Johansson LF, van Dijk F, de Boer EN et al. CoNVaDING: Single Exon Variation Detection in Targeted NGS Data. Human Mutation 2016; 37: 457–464.
