# Supplementary material for: Increased prevalence of kidney cysts in individuals carrying heterozygous COL4A3 or COL4A4 pathogenic variants
Source: Nephrol Dial Transplant. 2024 Feb 5;39(9):1442–8. doi: 10.1093/ndt/gfae031 (PMC11361806; doi:10.1093/ndt/gfae031)
Supplement: gfae031_Supplemental_Files [file gfae031_supplemental_files.zip › S3_Supplementary_ able_by_strata_of_age_eGFR .docx]

**Supplementary Table S3: Number and percentage of individuals with the number of kidney cysts (KC) by strata of age (A) and chronic kidney disease category (B)**

A)

| **Age** | **<29** | **30-39** | **40-49** | **50-59** | **60-69** | **>70** | **Total** |  |  |
| --- | --- | --- | --- | --- | --- | --- | --- | --- | --- |
| **Patients (n)** | **14** | **33** | **37** | **42** | **23** | **8** | **157** |  |  |
| **No KC (n)** | 11  (78.5%) | 22  (66.6%) | 20  (54.1%) | 17  (40.48%) | 4  (17.4%) | 0  0% | **74**  **(47.13%)** |  |  |
| **One kidney cyst (n)** | 2  (14.2%) | 3  (9.1%) | 7  (18.9%) | 4  (9.5%) | 3  (13%) | 3  (37.5%) | **22**  **(14.01%)** |  |  |
| **Two KC (n)** | 0  0% | 4  (12.1%) | 4  (10.81%) | 3  (7.14%) | 2  (8.69%) | 1  (12.5%) | **14**  **(8.92%)** |  |  |
| **Three or more KC (n)** | 1  (7.1%) | 4  (12.1%) | 6  (16.21%) | 18  (42.86%) | 14  (60.86%) | 4  (50%) | **43**  **(27.39%)** |  |  |

B)

| **Chronic kidney disease**  **category** | **G1** | **G2** | **G3a** | **G3b** | **G4** | **G5 no**  **dialysis** | **Total** |
| --- | --- | --- | --- | --- | --- | --- | --- |
| **Patients (n)** | **58** | **38** | **19** | **23** | **14** | **5** | **157** |
| **No KC (n)** | 37  (63.79%) | 22  (57,89%) | 4  (21,1%) | 6  (26.08%) | 4  (28,57%) | 1  (20%) | **74**  **(47,13%)** |
| **One kidney cyst (n)** | 7  (12.06%) | 8  (21.05%) | 4  (21,1%) | 1  (4.34%) | 1  (7.14%) | 0  (0%) | **21**  **(13.37%)** |
| **Two KC (n)** | 5  (8.62%) | 2  (5.26%) | 2  (10,5%) | 5  (21.73%) | 0  (0%) | 0  (0%) | **14**  **(8.92%)** |
| **Three or more KC (n)** | 8  (13.79%) | 6  (15.78%) | 9  (47,4%) | 13  (47.82%) | 9  (64,28%) | 4  (80%) | **47**  **(29.93%)** |
